# Impact of Neuron-Derived HGF on c-Met and KAI-1 in CNS Glial Cells: Implications for Multiple Sclerosis Pathology

**DOI:** 10.3390/ijms252011261

**Published:** 2024-10-19

**Authors:** Takuma Takano, Chie Takano, Hiroshi Funakoshi, Yoshio Bando

**Affiliations:** 1 Department of Functional Anatomy and Neuroscience, Asahikawa Medical University, Asahikawa 078-8510, Japan; 2 Department of Neurosurgery, Asahikawa Medical University, Asahikawa 078-8510, Japan; 3 Department of Advanced Medical Science, Asahikawa Medical University, Asahikawa 078-8510, Japan; 4 Department of Anatomy, Akita University Graduate School of Medicine, Akita 010-08543, Japan

**Keywords:** hepatocyte growth factor, glia, demyelination, myelin, EAE

## Abstract

Demyelination and axonal degeneration are fundamental pathological characteristics of multiple sclerosis (MS), an inflammatory disease of the central nervous system (CNS). Although the molecular mechanisms driving these processes are not fully understood, hepatocyte growth factor (HGF) has emerged as a potential regulator of neuroinflammation and tissue protection in MS. Elevated HGF levels have been reported in MS patients receiving immunomodulatory therapy, indicating its relevance in disease modulation. This study investigated HGF’s neuroprotective effects using transgenic mice that overexpressed HGF. The experimental autoimmune encephalomyelitis (EAE) model, which mimics MS pathology, was employed to assess demyelination and axonal damage in the CNS. HGF transgenic mice showed delayed EAE progression, with reduced CNS inflammation, decreased demyelination, and limited axonal degeneration. Scanning electron microscopy confirmed the preservation of myelin and axonal integrity in these mice. In addition, we explored HGF’s effects using a cuprizone-induced demyelination model, which operates independently of the immune system. HGF transgenic mice exhibited significant protection against demyelination in this model as well. We also investigated the expression of key HGF receptors, particularly c-Met and KAI-1. While c-Met, which is associated with increased inflammation, was upregulated in EAE, its expression was significantly reduced in HGF transgenic mice, correlating with decreased neuroinflammation. Conversely, KAI-1, which has been linked to axonal protection and stability, showed enhanced expression in HGF transgenic mice, suggesting a protective mechanism against axonal degeneration. These findings underscore HGF’s potential in preserving CNS structure and function, suggesting it may be a promising therapeutic target for MS, offering new hope for mitigating disease progression and enhancing neuroprotection.

## 1. Introduction

Multiple sclerosis (MS) is a chronic inflammatory disorder of the central nervous system (CNS), characterized by CNS inflammation, demyelination, and neurodegeneration. It is believed to result from the disruption of immunological self-tolerance to CNS myelin [1,2,3,4]. While glial cells such as astrocytes, microglia, and oligodendrocytes have been implicated in the pathogenesis of MS [4,5,6], the molecular mechanisms underlying MS pathology remain incompletely understood.

Understanding the multifaceted role of hepatocyte growth factor (HGF) in neuroprotection and repair across various neurodegenerative diseases is paramount for advancing therapeutic interventions. Originally isolated from hepatocytes, HGF is a cytokine with pleiotropic effects, significantly influencing immunoregulation and inflammation [7,8,9,10,11]. Accumulating evidence underscores its crucial involvement in neural processes, encompassing neurogenesis, neurite outgrowth, synaptic plasticity, neuronal survival, and repair [7]. By binding to c-Met, a receptor tyrosine kinase (RTK) also known as an HGF receptor, abundantly expressed in neural tissues, HGF initiates intricate intracellular signaling pathways that regulate neural cell proliferation, migration, and differentiation [9,11,12]. Moreover, KAI-1/CD82 (KAI-1), recognized as a metastasis suppressor, exhibits relevant neural contexts, influencing neural cell adhesion, neurite guidance, and synaptogenesis, thereby potentially interacting with c-Met signaling pathways [11,12]. Thus, HGF, acting through its receptor c-Met, not only orchestrates neural cell behaviors but also interacts with KAI-1, suggesting a more intricate regulatory network in neural processes.

Furthermore, several animal models have provided crucial insights into the therapeutic potential of HGF. Cerebrospinal fluid (CSF) levels of HGF in MS patients correlate negatively with disease activity, suggesting its potential as a biomarker and therapeutic target [13]. Studies in animal models of neurodegenerative diseases such as MS, Alzheimer’s disease, amyotrophic lateral sclerosis (ALS), cerebral ischemia, and spinal cord injury (SCI) have also demonstrated the beneficial effects of HGF [14,15,16,17,18]. In the context of MS, for instance, HGF plays a pivotal role in modulating inflammation. Previous studies have illuminated its capacity to alter the balance between pro-inflammatory (T helper cells 1 (Th1) and Th17) and anti-inflammatory (Th2 and Treg) CD4^+^ T cells in experimental autoimmune encephalomyelitis (EAE), a model for human MS [19]. HGF has been shown to exert an anti-inflammatory effect through the generation of tolerogenic dendritic cells, suppressing auto-reactive Th1 and Th17 cells, thereby reducing CD4^+^ T-cell-mediated nervous system injury in EAE models [19].

Beyond its influence on neural processes and inflammation, HGF plays a pivotal role in modulating other bodily systems. Notably, clinical trials focusing on ALS patients and individuals with acute SCI have unveiled promising outcomes following the intrathecal administration of human HGF [17,18]. HGF’s ability to inhibit CNS autoimmunity by inducing tolerogenic dendritic cells and regulatory T cells further underscores its therapeutic potential in neurodegenerative diseases [19]. Despite these advancements, the precise molecular mechanisms underlying its neuroprotective effects in the CNS remain elusive.

Among the several murine models of MS, EAE (experimental autoimmune encephalomyelitis) and cuprizone (CPZ) models are representative. Both models replicate distinct pathological aspects of the disease, which justifies their combined use in this study. The EAE model is widely used to study immune-mediated autoimmune reactions, particularly focusing on inflammation and the immune system’s involvement. It is an appropriate model for mimicking the relapsing–remitting form of MS, where immune responses play a significant role. On the other hand, the CPZ model primarily focuses on demyelination and oligodendrocyte injury, making it useful for studying the neurodegenerative and demyelinating mechanisms seen in MS. Given that MS is a multifactorial disease involving immune system dysregulation, inflammation, neurodegeneration, and demyelination, a single animal model cannot fully represent the complexity of its pathophysiology. By utilizing both EAE and CPZ models, we can comprehensively assess different pathological mechanisms—namely, immune inflammation and demyelination—providing a more integrated understanding of MS. In this study, the combination of these models allowed us to investigate both inflammatory responses and demyelination processes, thereby offering deeper insights into MS disease progression.

This study endeavored to bridge this gap by unraveling the molecular intricacies of HGF-mediated neuroprotection through the c-Met/KAI-1 signaling pathways. Such insights are indispensable for identifying novel therapeutic targets and developing effective treatments for neurodegenerative diseases, thereby offering hope for patients afflicted with these debilitating conditions.

## 2. Results

### 2.1. HGF Ameliorates the Progression of EAE

**Figure 1:**
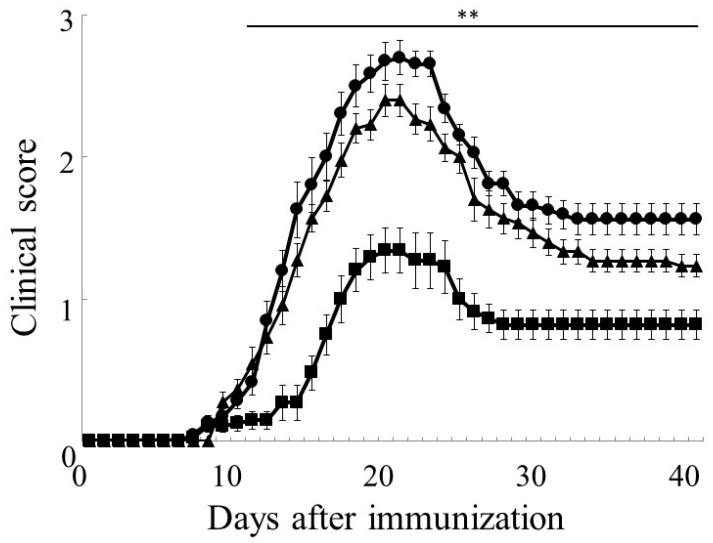
HGF can ameliorate MOG_35–55_-induced EAE. The progression of MOG_35–55_-induced EAE (EAE) was monitored daily and scored on disease severity on a clinical scale from 0 to 5, as described in the text. EAE symptoms in HGF-Tg+/+ mice (closed square, *n* = 24) were significantly milder than those in WT (closed circle, *n* = 23) and HGF-Tg+/− mice (closed triangle, *n* = 23). The mean clinical score (±SEM) is shown. ** *p* < 0.01 is shown (vs. WT).

Female C57BL/6J mice including HGF-Tg+/+, HGF-Tg+/−, and WT were immunized with MOG_35–55_ peptide to induce EAE. The clinical score was monitored daily. As shown in Figure 1 and Table 1, WT mice showed typical clinical symptoms by EAE, likely to peak disease at days 16–20. While HGF-Tg+/− mice showed a similar progression of EAE to WT mice, HGF-Tg+/+ mice showed a significant delay in onset and milder EAE (Figure 1 and Table 1). Since there was no significant difference between WT and HGF-Tg+/− mice, subsequent experiments were basically comparative between WT and HGF-Tg+/+ mice.

### 2.2. HGF Reduces Abnormal Myelin Morphology and Axonal Pathology in EAE

**Figure 2:**
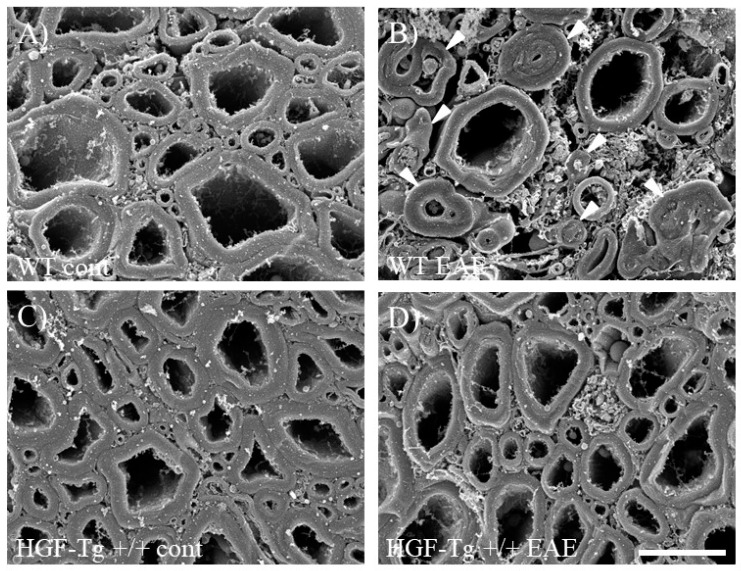
Myelin morphology in EAE mice. Myelin morphology in naïve and EAE mice was observed by SEM with the osmium-maceration method. The white matter of the lumbar spinal cord is shown. In naïve mice (cont), no difference in myelin morphology between WT (**A**) and HGF-Tg+/+ mice (**C**) was observed. While abnormal myelin morphology was markedly observed in the EAE spinal cord of WT (**B**), myelin morphology was almost preserved as normal in HGF-Tg+/+ mice (**D**). Representative data are shown (*n* = 5). Arrows indicate abnormal myelin morphologies as described previously [6,20]. Scale bar: 6 μm.

We have previously established the powerful technique of osmium-maceration SEM to observe morphological changes of myelin during demyelination [6,20,21]. This method enables us to observe abnormal myelin morphologies from the initial step of demyelination (detachment of myelin with compact lamination from the axon) to the formation of multiple and obstructive myelin [6,20,21]. In the white matter of the lumbar spinal cord in naïve mice, myelin morphology was no different between WT and HGF-Tg+/+ mice (Figure 2A,C). While various types of abnormal myelin morphologies were observed in the white matter of the EAE spinal cord in the WT mice (Figure 2B, arrowheads), HGF-Tg+/+ mice inhibited EAE-induced abnormal myelin formation at the initial step of demyelination, suggesting a nearly normal morphology (Figure 2D).

### 2.3. HGF Suppresses Inflammatory-Induced Demyelination and Axonal Degeneration During EAE

**Figure 3:**
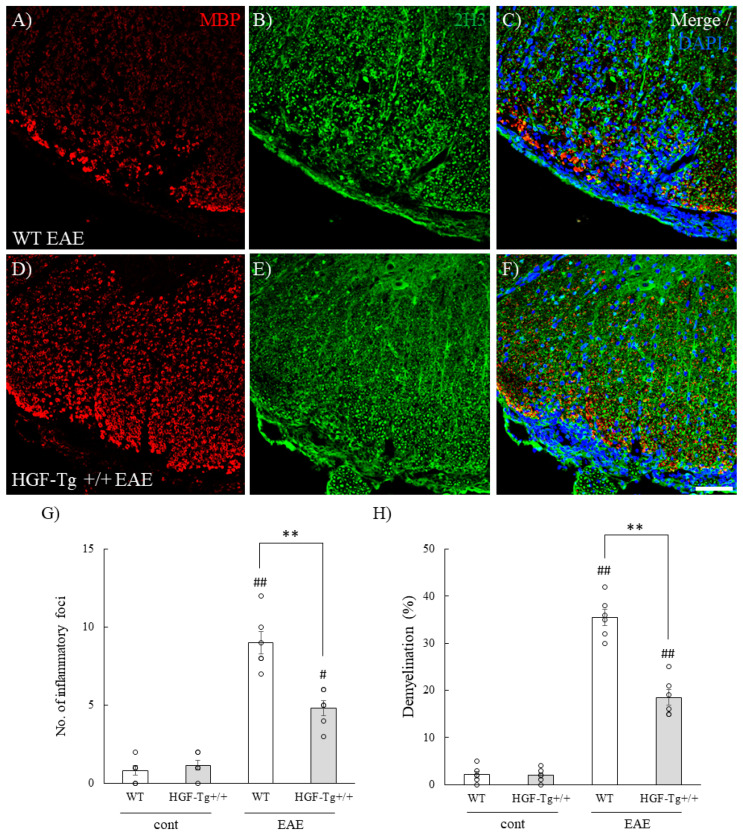
Suppression of EAE-induced demyelination and axonal injury in HGF-Tg+/+ mice. Frozen cross-sections of the lumbar spinal cords from either WT (**A**–**C**) or HGF-Tg+/+ mice (**D**–**F**) taken on day 20 post-immunization (peak disease) were stained with DAPI (blue, **C**,**F**), anti-MBP antibody (red, **A**,**D**,**C**,**F**), or anti-2H3 antibody (green, **B**,**E**,**C**,**F**). Representative data are shown. In panel (**A**), the intensity of MBP reactivity was decreased, indicating demyelination. Quantitative analysis (*n* = 5). The number of inflammatory foci (**G**) and % demyelination (**H**). Scale bars: 20 μm (**A**–**F**). # *p* < 0.05, ## *p* < 0.01 (vs. naïve), ** *p* < 0.01 (vs. WT).

**Figure 4:**
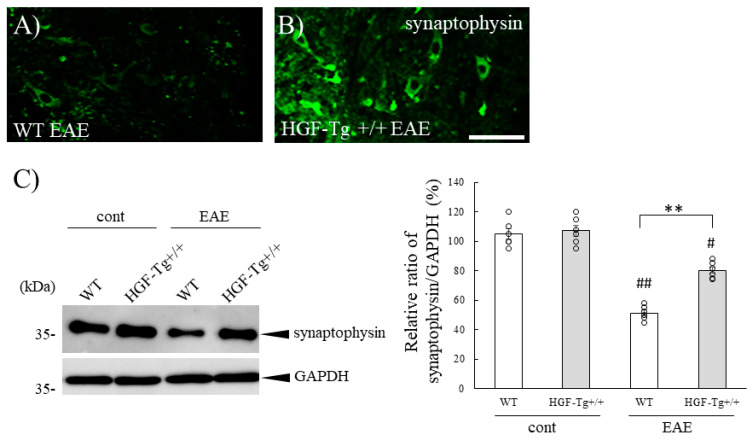
The expression level of synaptophysin is maintained in the EAE lumbar spinal cord of HGF-Tg+/+ mice but not in WT mice. Frozen cross-sections of the lumbar spinal cords from either WT (**A**) or HGF-Tg+/+ mice (**B**) taken on day 20 post-immunization (peak disease) were stained with anti-synaptophysin antibody (green). (**C**) Immunoblot analysis showing expression of synaptophysin in the lumbar spinal cords from naïve and EAE mice. GAPDH was used as a loading control. Representative data are shown (*n* = 5). The ratio of synaptophysin/GAPDH is shown. Scale bars: 50 μm. # *p* < 0.05, ## *p* < 0.01 (vs. naïve), ** *p* < 0.01 (vs. WT).

To examine the neuroprotective effect of HGF on EAE-induced demyelination and axonal degeneration, immunohistochemistry was then performed at the lumbar levels of spinal cords in EAE mice (Figure 3). The number of DAPI-positive cells at inflammatory sites reflected the approximate estimation of inflammatory foci (see the Osmium-Maceration SEM Analysis and Immunohistochemistry Section in the Materials and Methods Section [6,21,22,23,24,25]). DAPI staining demonstrated peripheral inflammatory cells invaded the white matter of the EAE-lumbar spinal cord of EAE-WT mice (Figure 3C). On the other hand, HGF-Tg+/+ mice clearly showed that the distribution of peripheral inflammatory cells was restricted on the pia matter (Figure 3F,G). MBP staining (myelin staining [6,21]) also clearly demonstrated that the intensity of MBP reactivity in HGF-Tg+/+ mice was kept relative to both the WT and HGF+/− EAE mice, suggesting that HGF could suppress demyelination in the EAE spinal cord (Figure 3A,D,H). In addition, 2H3 staining (a marker of neurofilament [26]) in HGF-Tg+/+ mice showed similar result with reduced axonal degeneration in comparison with WT mice (Figure 3B,E,H). HGF reduced CNS inflammation by suppressing the infiltration of inflammatory cells from the periphery (Figure 3C,F). In HGF-Tg+/− mice, peripheral inflammatory cells were observed in the blood vessels but not in the white matter of the lumbar spinal cord, and the results obtained from MBP and 2H3 staining seemed to indicate a dose dependency of HGF. Moreover, the expression of synaptophysin in the motor neurons of the spinal ventral horn revealed a decrease in synaptophysin expression in EAE-induced WT mice (Figure 4A). Conversely, in HGF-Tg+/+ mice, the reduction in synaptophysin expression induced by EAE was suppressed (Figure 4B,C). These results indicated that HGF produced by neurons suppressed EAE-induced demyelination and axonal degeneration.

### 2.4. HGF Reduces Inflammatory Demyelination and Oligodendroglial Cell Death During EAE

**Figure 5:**
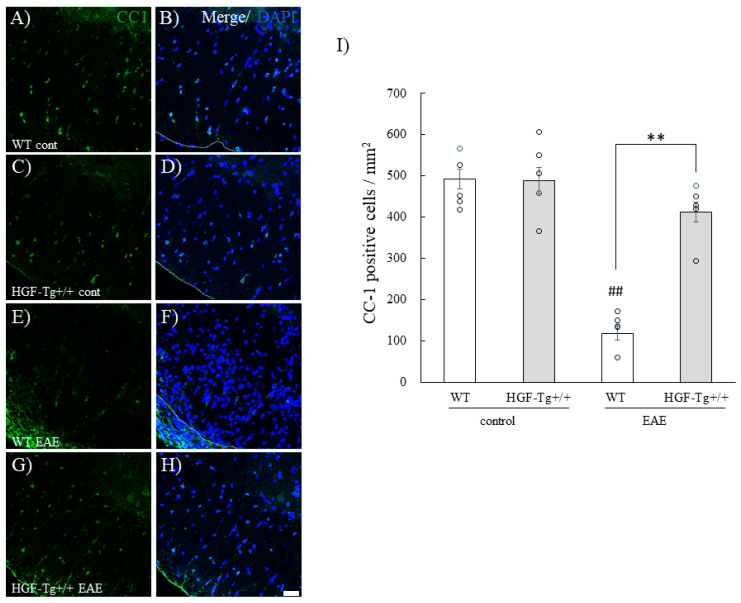
Suppression of EAE-induced oligodendroglial cell death in HGF-Tg+/+ mice. Frozen cross-sections of the lumbar spinal cords from either WT (**A**,**B**,**E**,**F**) or HGF-Tg+/+ mice (**C**,**D**,**G**,**H**) taken on day 0 (**A**–**D**) and day 20 post-immunization (peak disease; **E**–**H**) were stained with anti-CC1 antibody (green, **A**,**C**,**E**,**G**) and DAPI (blue, **B**,**D**,**F**,**H**). Quantitative analysis of the number of CC-1^+^ oligodendrocytes (**I**). Representative data are shown (*n* = 5). Scale bar: 20 μm. ## *p* < 0.01 (vs. naïve), ** *p* < 0.01 (vs. WT).

Oligodendroglial cell death was observed during EAE. The number of oligodendrocytes the EAE spinal cord was then examined by immunohistochemistry with anti-CC1 antibody (a marker of mature oligodendrocyte [21]). In the white matter of the lumbar spinal cord of naïve mice, immunohistochemistry exhibited that the number of oligodendrocytes was no different between WT mice (Figure 5A) and HGF-Tg+/+ mice (Figure 5C,I). While the number of CC1^+^ oligodendrocytes in the EAE lumbar spinal cord of WT mice was decreased (Figure 5E), HGF-Tg+/+ mice showed suppression of oligodendroglial cell death during EAE (Figure 5G,I).

### 2.5. HGF Reduces Astrocytic and Microglial Gliosis/Activation During EAE

**Figure 6:**
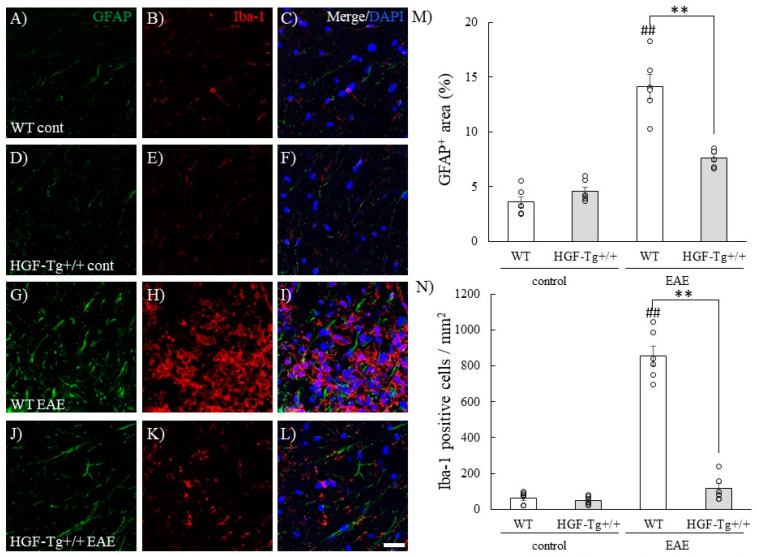
HGF attenuates GFAP^+^ astrocytes and Iba-1^+^ microglia/macrophages in EAE. Frozen cross-sections of the lumbar spinal cords from either WT (**A**–**C**,**G**–**I**) or HGF-Tg+/+ mice (**D**–**F**,**J**–**L**) taken on day 0 (**A**–**F**) and day 20 post-immunization (peak disease; **G**–**L**) were stained with anti-GFAP antibody (green, **A**,**D**,**G**,**J**) and anti-Iba-1 antibody (red, **B**,**E**,**H**,**K**). Merge images are also shown with DAPI staining (blue, **C**,**F**,**I**,**L**). Quantitative analysis of the number of GFAP^+^ area (**M**) and the number of Iba-1^+^ microglia/macrophages (**N**). Representative data are shown (*n* = 6). Scale bars: 20 μm. ## *p* < 0.01 (vs. naïve), ** *p* < 0.01 (vs. WT).

Astrocytes and microglia were also associated with the EAE pathogenesis [21,24]. Both astrocytic and microglial gliosis during EAE were then examined. The numbers of GFAP-positive astrocytes and Iba-1-positive microglia/macrophages in the white matter of the spinal cord were increased by EAE induction in WT mice (Figure 6A–C,G–I,M,N). By contrast, HGF suppressed astrocytic/microglial gliosis during EAE (Figure 5D–F,J–N). These observations suggest that HGF suppresses an increasing number of astrocytes and microglia in the white matter of the spinal cord during EAE.

### 2.6. c-Met, an HGF Receptor, Is Expressed in Astrocytes, Microglia, and Vascular Endothelial Cells During EAE

**Figure 7:**
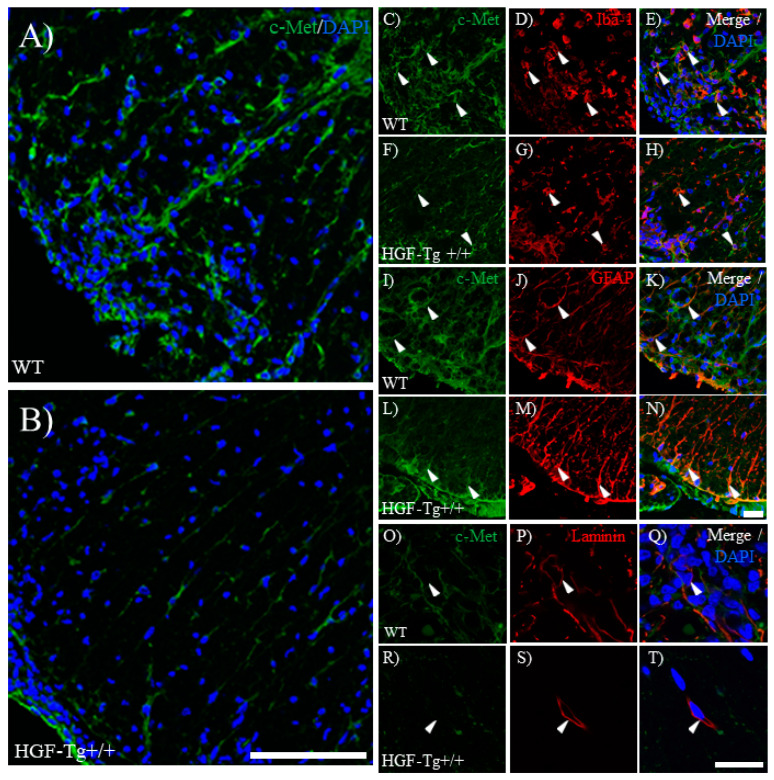
Expression of c-Met on astrocytes and microglia in EAE. Frozen cross-sections of the lumbar spinal cords from either WT (**A**,**C**–**E**,**I**–**K**,**O**–**Q**) or HGF-Tg+/+ mice (**B**,**F**–**H**,**L**–**N**,**R**–**T**) taken on day 20 post-immunization (peak disease) were stained with anti-c-Met antibody (green, **A**–**C**,**F**,**I**, **L**,**O**,**R**) and either anti-Iba-1 antibody (red, **D**,**G**), anti-GFAP antibody (red, **J**,**M**), or anti-Laminin antibody (red, **P**,**S**). Merge images are also shown with DAPI staining (blue) (**A**,**B**,**E**,**H**,**K**,**N**,**Q**,**T**). Arrows indicate co-expressing cells. Representative data are shown (*n* = 5). Scale bars: 50 μm (**A**,**B**), 20 μm (**C**–**N**), and 10 μm (**O**–**T**).

**Figure 8:**
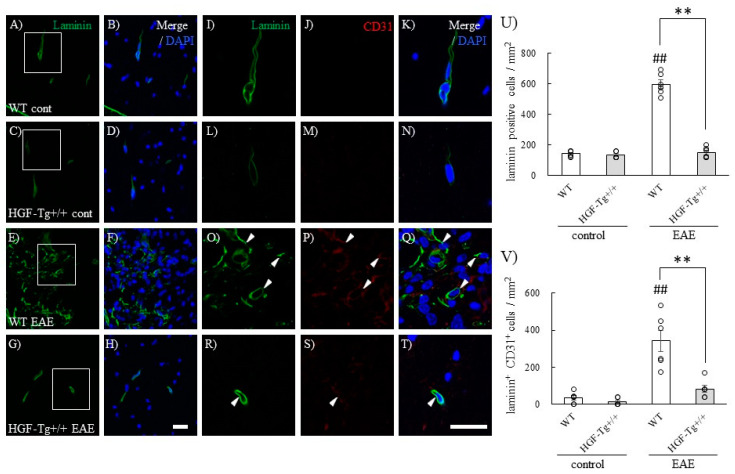
HGF suppresses EAE-induced angiogenesis. Frozen cross-sections of the lumbar spinal cords from either WT (**A**,**B**,**E**,**F**,**I**–**K**,**O**–**Q**) or HGF-Tg+/+ mice (**C**,**D**,**G**,**H**,**L**–**N**,**R**–**T**) taken on day 0 (**A**–**D**,**I**–**N**) or day 20 post-immunization (peak disease, **E**–**H**,**O**–**T**) were stained with anti-Laminin antibody (green, **A**,**C**,**E**,**G**,**I**,**L**,**O**,**R**) and anti-CD31 antibody (red, **J**,**M**,**P**,**S**). Merge images are also shown with DAPI staining (blue) (**B**,**D**,**F**,**H**,**K**,**N**,**Q**,**T**). High-magnification images of the inset in (**A**,**C**,**E**,**G**) are shown in (**I**–**K**,**L**–**N**,**O**–**Q**,**R**–**T**), respectively. Representative data are shown (*n =* 6). Scale bars: 50 μm (**A**,**B**), 20 μm (**C**–**N**), and 10 μm (**O**–**T**). Quantitative analysis of the numbers of Laminin^+^ cells (**U**) and Laminin^+^ CD31^+^ cells (**V**) in the spinal cords of naïve and EAE mice. ## *p* < 0.01 (vs. naïve), ** *p* < 0.01 (vs. WT).

The expression of the HGF receptor c-Met was next examined in the EAE spinal cord. As shown in Figure 7, immunohistochemistry demonstrated that the higher expression of c-Met was observed in the EAE spinal cord of WT mice (Figure 7A) rather than HGF-Tg+/+ mice (Figure 7B). In addition, c-Met was expressed in Iba-1^+^ microglia/macrophages (Figure 7C–H), GFAP^+^ astrocytes (Figure 7I–N), and laminin^+^ vascular endothelial cells (Figure 7O–T) in the white matter of the spinal cord of both WT and HGF-Tg+/+ mice (Figure 7, arrowheads). Since vascular alterations such as angiogenesis are implicated in the pathogenesis of EAE [21,27], we further examined angiogenesis induced by EAE in these mice. CD31, which is expressed in the endothelial cells of blood vessels, can be used specifically as a representative marker for angiogenesis [6,21,27,28,29]. In the current study, CD31 is used as a marker for angiogenesis. Although there was no difference in laminin^+^ cells and laminin^+^CD31^+^ cells in naïve conditions between WT (Figure 8A,B,I–K) and HGF-Tg+/+ mice (Figure 8C,D,L–N), immunohistochemistry showed an increased number of laminin^+^ cells and angiogenic vessels (laminin^+^CD31^+^ cells) in the white matter of the EAE spinal cords of WT mice (Figure 8E,F,O–Q, arrowheads) but not in those of HGF-Tg+/+ mice (Figure 8G,H,R–T,U,V, arrowheads). These observations indicate that EAE-induced angiogenesis is associated with worsening EAE pathology and that HGF plays an important role in regulating EAE pathogenesis by suppressing EAE-induced angiogenesis.

### 2.7. HGF Reduces Glutamate Toxicity by Maintaining EAAT2 Expression in Astrocytes

**Figure 9:**
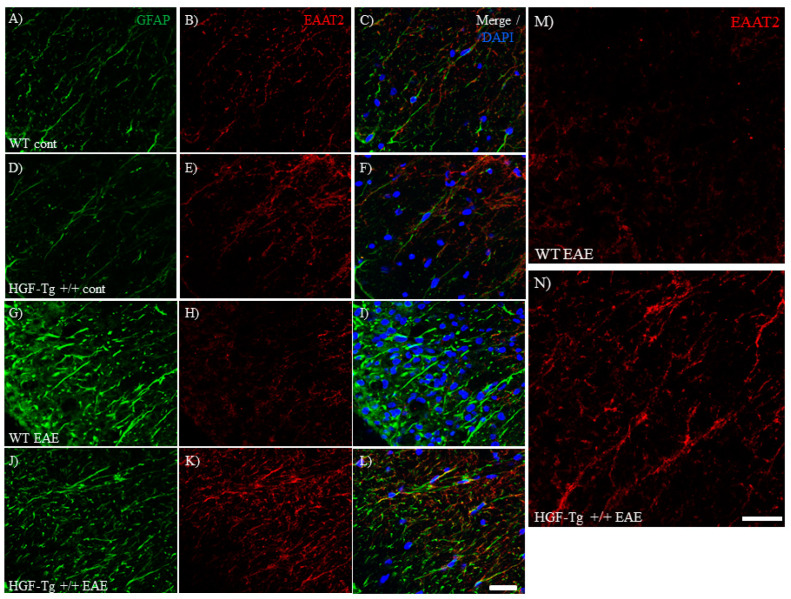
Expression of EAAT2 on astrocytes is sustained in HGF-Tg+/+ mice. Frozen cross-sections of the lumbar spinal cords from either WT (**A**–**C**,**G**–**I**,**M**) or HGF-Tg+/+ mice (**D**–**F**,**J**–**L**,**N**) taken on day 0 (**A**–**F**) or day 20 post-immunization (peak disease, **G**–**N**) were stained with anti-GFAP antibody (green, **A**,**D**,**G**,**J**) and anti-EAAT2 antibody (red, **B**,**E**,**H**.**K**). Merge images are also shown with DAPI staining (blue) (**C**,**F**,**I,L**). High-magnification images of the insets in **H** and **K** are shown in **M** and **N**, respectively. Representative data are shown (*n* = 5). Scale bars: 50 μm (**A**–**L**) and 20 μm (**M**,**N**).

Glutamate toxicity is the major cause of the pathogenesis of EAE [21,30,31]. Previous studies have also demonstrated that HGF can reduce glutamate toxicity in murine animal disease models [12]. To assess the effect of HGF on glutamate toxicity, the distribution and expression of EAAT2, a glutamate transporter in astrocytes, was next explored at the peak of the EAE disease. In naïve conditions, EAAT2 was expressed in GFAP-positive astrocytes in the white matter of the lumbar spinal cord in both WT (Figure 9A–C) and HGF-Tg+/+ mice (Figure 9D–F). Although the decreased expression level of EAAT2 in astrocytes at peak disease of EAE was observed in WT mice (Figure 9G–I,M), HGF-Tg+/+ mice showed a sustained higher expression of EAAT2 at peak disease of EAE (Figure 9J–L,N). These results indicate that HGF reduces glutamate toxicity by sustainable expression of astrocytic EAAT2 to remove glutamate in the white matter of the EAE spinal cord.

### 2.8. HGF Regulates EAAT2 Expression by HGF/c-Met Signaling in Astrocytes

**Figure 10:**
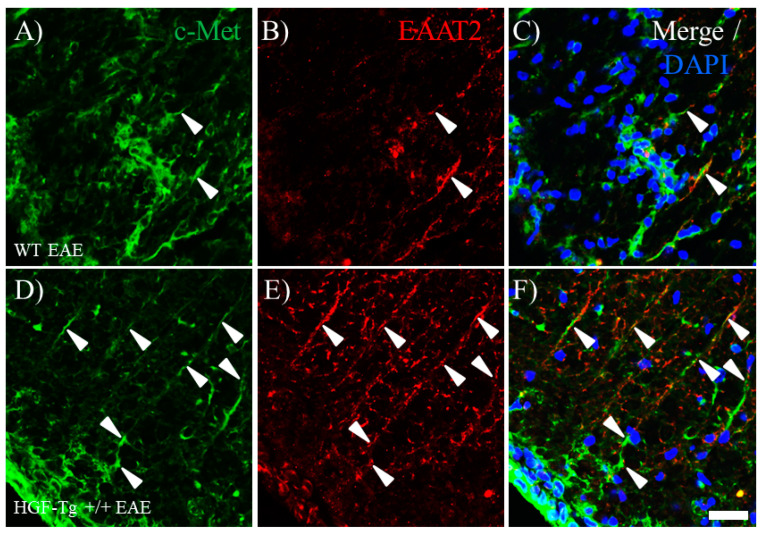
Co-expression of c-Met and EAAT2 in EAE. Frozen cross-sections of the lumbar spinal cords from either WT (**A**–**C**) or HGF-Tg+/+ mice (**D**–**F**) taken on day 20 post-immunization (peak disease) were stained with anti-c-Met antibody (green, **A**,**D**) and anti-EAAT2 antibody (red, **B**,**E**). Merge images are also shown with DAPI staining (blue, **C**,**F**). Arrows indicate c-Met and EAAT2 co-expressing astrocytes. Representative data are shown (*n* = 5). Scale bars: 20 μm (**A**–**F**).

**Figure 11:**
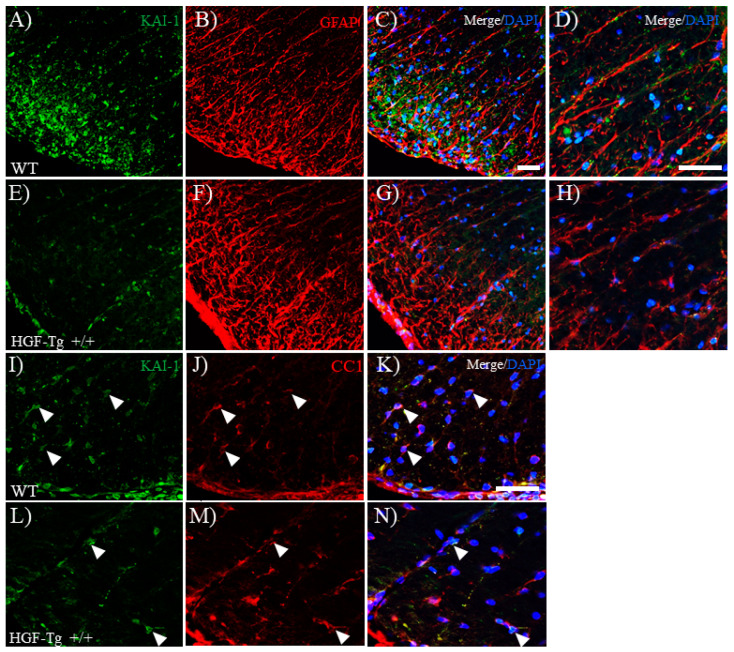
KAI-1 expression on astrocytes and oligodendrocytes in EAE. Frozen cross-sections of the lumbar spinal cords from either WT (**A**–**D**,**I**–**K**) or HGF-Tg+/+ mice (**E**–**H**,**L**–**N**) taken on day 20 post-immunization (peak disease) were stained with anti-KAI-1 antibody (green, **A**,**E**,**I**,**L**) and either anti-GFAP antibody (red, **B**,**F**) or anti-CC1 antibody (red, **J**,**M**). Merge images are also shown with DAPI staining (blue) (**C**,**D**,**G**–**H**,**K**,**N**). High-magnification images of **C** and **G** are shown in **D** and **H**, respectively. Arrows indicate KAI-1-expressing oligodendrocytes. Representative data are shown (*n* = 5). Scale bars: 50 μm (**A**–**G**) and 20 μm (**D**,**H**,**I**–**N**).

To examine the possibility of the effect of HGF/c-Met signaling on the induction of astrocytic EAAT2 expression, we further investigated the c-Met expression in EAAT2^+^ astrocytes. Immunohistochemistry demonstrated that most EAAT2-expressing astrocytes also expressed c-Met (Figure 10). As shown in Figure 9, HGF-Tg+/+ mice showed a sustained higher expression of EAAT2 in the white matter of the EAE spinal cord (Figure 10E), compared with WT mice (Figure 10B). On the other hand, interestingly, increased c-Met expression was observed in the EAE spinal cords of WT mice (Figure 10A) rather than HGF-Tg+/+ mice (Figure 10D). It appeared that most cells expressing EAAT2^+^ astrocytes also co-expressed c-Met. Assuming that WT mice have more exacerbated inflammation than HGF-Tg+/+ mice, the persistent high expression of c-Met/EAAT2 in HGF-Tg+/+ mice would lead to lower glutamate toxicity during EAE. In contrast, KAI-1 expression on astrocytes was lower in WT mice (Figure 11A–D) and higher in HGF-Tg mice (Figure 11E–H). Moreover, KAI-1 expression was also observed in the CC1^+^ oligodendrocyte in WT (Figure 11I–K) but not that in HGF-Tg+/+ mice (Figure 11L–N). These results indicate that the HGF/c-Met/KAI-1 signaling pathways critically regulate EAE pathology.

### 2.9. HGF Also Suppressed CPZ-Induced Demyelination in Mice

We then next investigated the effect of HGF on demyelination in another CNS demyelination model. CPZ-induced demyelination, which is almost independent of the immune system to cause demyelination, was applied [20,32,33]. CPZ is a chelator of copper that is added to the chow. CPZ slowly induced demyelination by oligodendroglial cell death during 4–6 weeks after treatment mainly in the corpus callosum of mouse brain. 

**Figure 12:**
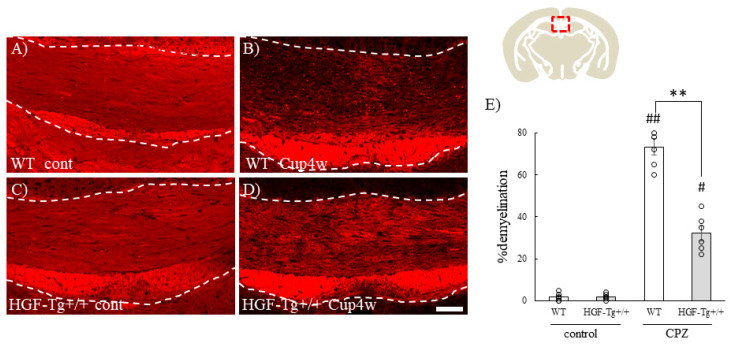
Suppression of CPZ-induced demyelination in HGF-Tg+/+ mice. Frozen cross-sections of corpus callosum from either WT (**A**,**B**) or HGF-Tg+/+ mice (**C**,**D**) taken on either day 0 (**A**,**C**) or 4 weeks of 0.2% CPZ administration (**B**,**D**) were stained with fluoromyelin. The corpus callosum, corresponding to the red box in the brain illustration, is shown. The corpus callosum is represented by white dashed lines. Representative data are shown (*n* = 6). Scale bars: 100 μm. (**E**) Quantitative analysis of the numbers of % demyelination in the corpus callosum of naïve and EAE mice (*n* = 6). # *p* < 0.05, ## *p* < 0.01 (vs. naïve), ** *p* < 0.01 (vs. WT).

In the current study, CPZ in the chow continued for 4 weeks, and then the mice were sacrificed to evaluate the brain. The corpus callosum was examined by histology to determine the effect of HGF on CPZ-induced demyelination. Fluoromyelin staining demonstrated that in naïve conditions, there was no difference between WT and HGF-Tg+/+ mice (Figure 12A,C). While severe demyelination, which was reflected by a decreased level of MBP staining, was observed in the corpus callosum of WT mice during CPZ-induced demyelination (Figure 12B), HGF-Tg+/+ mice showed suppression of demyelination in the corpus callosum (Figure 12E).

**Figure 13:**
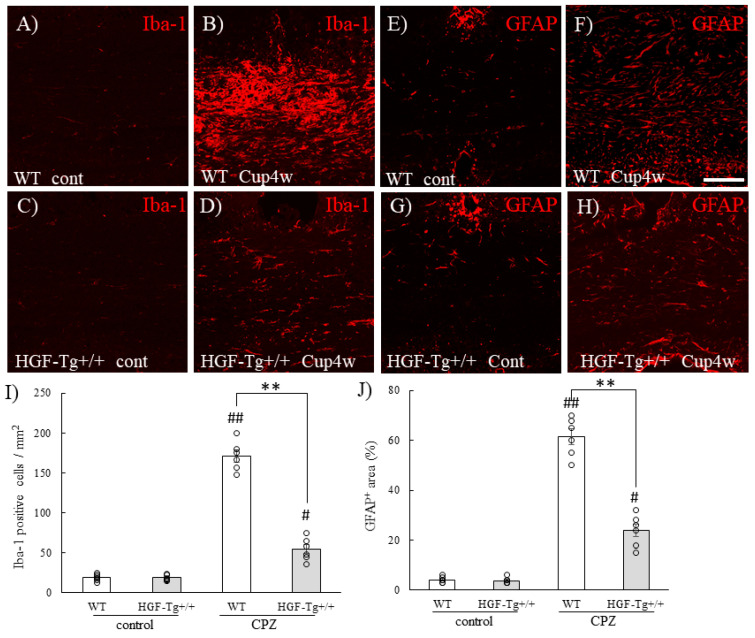
Expression of GFAP^+^ astrocytes and Iba-1^+^ microglia in CPZ-induced demyelination. Frozen cross-sections of corpus callosum from either WT (**A**,**B**,**E**,**F**) or HGF-Tg+/+ mice (**C**,**D**,**G**,**H**) taken on day 0 (**A**,**C**,**E**,**G**) or 4 weeks of 0.2% CPZ administration were stained with either GFAP (red, **A**–**D**) and Iba-1 (red, **E**–**H**). GFAP^+^ astrocytes and Iba-1^+^ microglia were activated in the corpus callosum of the CPZ-treated demyelination in WT but not that in HGF-Tg+/+ mice. Representative data are shown. Scale bars: 20 μm. Quantitative analysis of the numbers of Iba-1^+^ cells (**I**) and GFAP^+^ area (**J**) in the corpus callosum of naïve and EAE mice (*n* = 6). # *p* < 0.05, ## *p* < 0.01 (vs. naïve), ** *p* < 0.01 (vs. WT).

In the CPZ model, immune cells such as CD3-positive cells have also been reported to infiltrate the corpus callosum, but the number of immune cells (CD3-positive cells, 30 cells/mm^2^ [34]) is much smaller than the number of Iba-1-positive microglia (20 cells/mm^2^ in control, 170 cells/mm^2^ in cuprizone treatment; Figure 13, indicating that the influence of immune system cells from the periphery on the pathology is considered small and independent as previously described [20,32,33].

### 2.10. HGF Suppressed Gliosis in CPZ-Induced Demyelination

In this model, GFAP^+^ astrocytes and Iba-1^+^ microglia were accumulated in the corpus callosum in CPZ-treated mice, but the blood–brain barrier was not injured by CPZ [20,32,33]. Therefore, inflammatory cells from the periphery were not observed. As a result, Iba-1^+^ cells could be considered to be microglia. Consistent with previous studies [20,32,33], GFAP^+^ astrocytes and Iba-1^+^ microglia in WT mice were accumulated in the corpus callosum in CPZ-induced demyelination (Figure 13A–D,I). On the other hand, HGF-Tg+/+ mice showed that limited numbers of astrocytes and microglia were observed in the corpus callosum (Figure 13E–H,J). These results indicated that HGF suppressed demyelination by reducing gliosis/glial activation in CPZ-induced demyelination.

### 2.11. c-Met/KAI-1 Is Expressed on Astrocytes and Microglia in CPZ-Induced Demyelination

**Figure 14:**
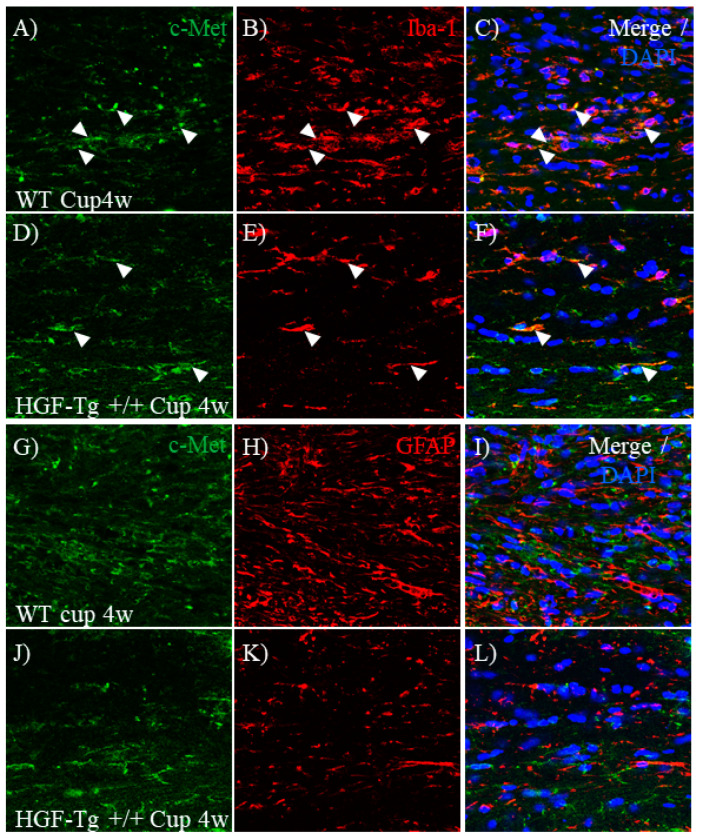
Expression of c-Met on astrocytes and microglia in CPZ-treated demyelination. Frozen cross-sections of corpus callosum from either WT (**A**–**C**,**G**–**I**) or HGF-Tg+/+ mice (**D**–**F**,**J**–**L**) taken on 4 weeks of 0.2% CPZ administration were stained with anti-c-Met antibody (**A**,**D**,**G**,**H**) and either anti-Iba-1 antibody (red, **B**,**E**) or anti-GFAP antibody (red, **H**,**K**). Merge images are also shown with DAPI staining (blue) (**C**,**F**,**I**,**L**). Arrows indicate c-Met-expressing Iba-1^+^ cells. Representative data are shown (*n* = 5). Scale bars: 20 μm.

**Figure 15:**
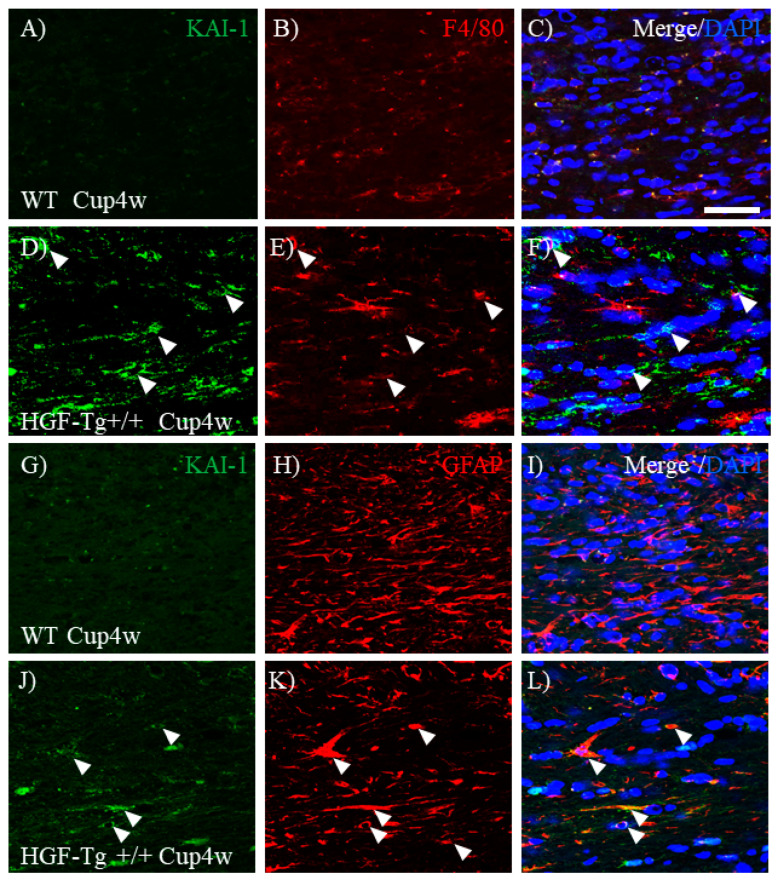
Expression of KAI-1 on astrocytes and microglia in CPZ-treated demyelination. Frozen cross-sections of corpus callosum from either WT (**A**–**C**,**G**–**I**) or HGF-Tg+/+ mice (**D**–**F**,**J**–**L**) taken on 4 weeks of 0.2% CPZ administration were stained with anti-KAI-1 antibody (**A**,**D**,**G**,**H**) and either anti-F4/80 antibody (red, **B**,**E**) or anti-GFAP antibody (red, **H**,**K**). Merge images are also shown with DAPI staining (blue) (**C**,**F**,**I**,**L**). Arrows indicate KAI-1-expressing GFAP^+^ cells. Representative data are shown (*n* = 5). Scale bars: 20 μm.

The expression of c-Met/KAI-1 was then examined in CPZ-induced demyelination. Immunohistochemistry revealed higher expression of c-Met in the corpus callosum of WT mice (Figure 14A) during CPZ-induced demyelination (Cup 4w) compared with HGF-Tg+/+ mice (Figure 14D). Consistent with the results obtained from the EAE model, c-Met was observed in Iba-1^+^ microglia (Figure 14A–F) and GFAP^+^ astrocytes (Figure 14G–L). However, c-Met expression in astrocytes was scarcely observed in the CPZ-treated corpus callosum of HGF-Tg+/+ mice. In contrast, immunohistochemistry demonstrated higher expression of KAI-1 in the corpus callosum of HGF-Tg+/+ mice (Figure 15D) during CPZ-induced demyelination compared with WT mice (Figure 15A). However, most cells expressing KAI-1 in the CPZ-treated corpus callosum were not F4/80-positive microglia but tended to be expressed in GFAP-positive astrocytes (Figure 15D–F,J–L). Conversely, higher expression of KAI-1 in F4/80^+^ microglia and GFAP^+^ astrocytes was observed in CPZ-treated HGF-Tg+/+ mice (Figure 15A–C,J–L, arrowheads) compared with CPZ-treated WT mice (Figure 15D–F,G–I).

## 3. Discussion

The results from the current study indicate that HGF provides significant neuroprotective benefits in experimental models of MS. HGF produced by neurons suppressed not only demyelination followed by axonal damage in EAE but also CPZ-induced demyelination, which is a completely different animal model. It suggests that neurons may have the ability to protect oligodendrocytes by producing neuroprotective factors such as HGF. In addition, the HGF/c-Met/KAI-1 signaling pathways in astrocytes and microglia played a critical role in neuroinflammation-mediated demyelination and axonal loss in MS.

In Figure 1, the delayed onset and reduced severity of EAE observed in HGF-Tg+/+ mice, characterized by sustained HGF supply from neurons, suggest the potential of early and continuous HGF delivery in suppressing EAE onset and progression. It has been confirmed that HGF production is sustained from postnatal 2 weeks in HGF-Tg+/+ mice [35]. These results also imply the promising prospect of administering HGF therapy during the remission. Similar to the current study, Benkhoucha et al. previously reported that HGF exerted an anti-inflammatory effect through the generation of tolerogenic dendritic cells with the consequent suppression of auto-reactive Th1 and Th17 cells and leading to the reduction in CD4^+^ T-cell-mediated nervous system injury in the EAE, in which HGF was overexpressed (HGF-Tg mice) [19].

While it has not been clear which cells are affected by HGF, the current study identified the cells expressing its main receptor, c-Met and KAI-1, which is a meaningful step forward in understanding its cellular targets. Our interpretations rely heavily on the results of immunohistochemical analysis, which are crucial for the validity of our findings. There may be a potential limitation due to some background signal in immunostaining. Nevertheless, we believe that our conclusions remain sound. We have taken care to ensure clear colocalization, as indicated by arrows, and the specific signals central to our findings remain distinguishable from any background. Further studies, including in vitro and flow cytometric analyses, will provide deeper insights into the pathological significance of HGF/c-Met/KAI-1 expression and its signaling pathway.

Our results suggest that neuron-derived HGF contributes to inflammation-associated glial cell accumulation (Figure 3, Figure 6 and Figure 13). This result was supported by histochemical analysis showing that HGF-Tg+/+ mice exhibited minimal inflammatory foci (Figure 3) and gliosis in the EAE spinal cord (Figure 6) and the CPZ-treated corpus callosum (Figure 13). HGF produced by neurons not only serves an endocrine function but also functions in a paracrine manner when released into the extracellular space by neurons. This paracrine mechanism potentially involves the action of HGF in non-neuronal cells surrounding the neurons.

While it cannot be ruled out that HGF may act on oligodendrocyte precursor cells to promote proliferation or be involved in induction of oligodendroglial differentiation [33,34], HGF-Tg+/+ mice exhibited that HGF suppressed both EAE-induced demyelination followed by axonal injury (Figure 3 and Figure 4) and CPZ-induced demyelination (Figure 12). CPZ induces demyelination by inducing cell death in a CNS-specific and oligodendrocyte-selective manner [20,32,33]. Additionally, EAE significantly reduced the number of CC-1^+^ oligodendrocytes in the spinal cords of WT mice but not in HGF-Tg+/+ mice (Figure 5). HGF also contributes to the suppression of demyelination and axonal degeneration via oligodendroglial cell death. Notably, SEM analysis provided further insight, highlighting that HGF-Tg+/+ mice displayed a remarkable suppression of EAE-induced ultrastructural pathological changes, particularly evident in preserving normal myelin morphology compared with WT mice (Figure 2). We have continuously demonstrated that our SEM technique is an invaluable tool in elucidating ultrastructural changes in myelin [6,20,21]. We have previously reported that oligodendrocytes and myelin sheaths can be neuroprotective by maintaining their structure and function [6,20,21]. These findings collectively suggest that neuron-derived HGF protects oligodendrocytes by promoting oligodendroglial cell survival in EAE- and CPZ-induced demyelination.

In the current study, we attempted to elucidate the molecular mechanism of the neuroprotective effect mediated by the c-Met/KAI-1 signaling pathways via HGF. HGF reduces pro-inflammatory cytokines and enhances anti-inflammatory cytokines. HGF has a high affinity for its cell surface receptors c-Met and KAI-1 [7]. While HGF has receptors such as c-Met and KAI-1, little is known about how the molecular mechanisms mediated by HGF-mediated c-Met/KAI-1 signaling pathways affect its neuroprotective effects in the CNS. As shown in Figure 7, c-Met was found to be expressed in astrocytes, microglia, and vascular endothelium. EAE-induced angiogenesis referred to as laminin/CD31 expression in the spinal cord was observed in WT but not in HGF-Tg+/+ mice (Figure 7 and Figure 8). These vascular endothelial cells expressing laminin/CD31 also expressed c-Met. EAE-induced angiogenesis may recruit inflammatory cell infiltration from the periphery and exacerbate inflammation. HGF-Tg+/+ mice demonstrated suppression of EAE-induced angiogenesis. This suggests that c-Met-mediated angiogenesis in EAE may be dependent on the degree of inflammation.

Interestingly, HGF-Tg+/+ mice also showed a decreasing level of EAE-induced astrocytic and microglial gliosis (Figure 6), resulting in favoring a reduction in glutamatergic neurotoxicity during the peak disease of EAE (Figure 7). In addition, the reduction in this gliosis might attenuate the expression levels of the predominant astrocytic glutamate transporter, EAAT2, in HGF-Tg mice (Figure 7). Astrocytes and microglia also implicate the progression of EAE by producing some inflammatory cytokines, which promote oligodendroglial cell death and demyelination [6,20,21,24]. These observations suggest that high concentrations of HGF resulted in low expression of c-Met and elevated expression of EAAT2, which may have resulted in mild gliosis and inflammation, whereas low concentrations of HGF resulted in elevated c-Met expression and low expression of EAAT2, which may have resulted in pronounced gliosis and increased glutamate toxicity. In other words, the expression of c-Met in astrocytes and microglia and the expression of EAAT2 in astrocytes may be HGF-dependent and correlated with the exacerbation of inflammation. Further studies in the HGF-mediated downstream signaling of c-Met and EAAT2 in astrocytes and microglia are required.

**Figure 16:**
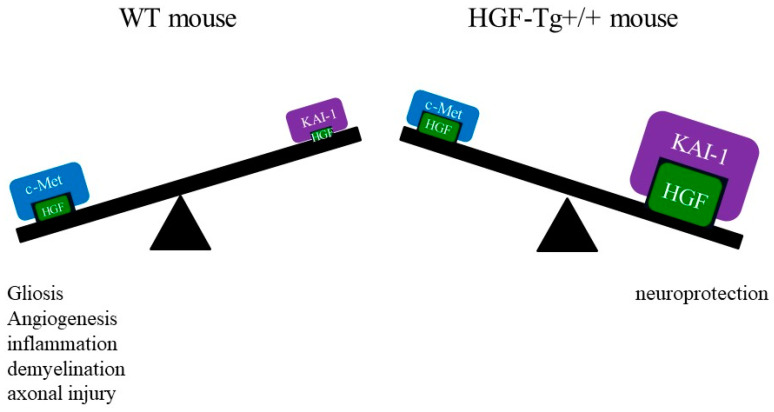
Schema of HGF/c-Met/KAI-1 signaling pathway. The HGF/c-Met signaling and the HGF/KAI-1 signaling play opposite roles.

We further investigated the expression and distribution of c-Met and KAI-1/CD82 and clarified that the changes in c-Met and KAI-1 expression were exactly opposite (Figure 16). The expression of c-Met in most of the EAAT2-positive astrocytes suggests that the HGF/c-Met signaling may work toward promoting gliosis and angiogenesis in EAE. By contrast, higher expression of KAI-1 was detected on GFAP^+^ astrocytes in the EAE spinal cords in HGF-Tg mice rather than in WT mice. These results were similar for the CPZ model. These observations suggest that the HGF/KAI-1 signaling pathways observed in these cells in HGF-Tg+/+ mice could suppress neuroinflammation during EAE. Importantly, c-Met expression is induced by inflammatory cytokines and has formed the basis for a crucial role for the HGF/c-Met signaling in the inflammatory response to tissue injury [7,36]. On the other hand, KAI-1 exerts inhibitory effects on the HGF-induced activation of c-Met. Consistent with our current results, it has been suggested that KAI-1 may act in the inhibitory role of the c-met pathway in the pathogenesis of cancer [36,37,38,39,40,41]. Taken together, our results indicate that the expressions of c-Met and KAI-1 possibly reflect the degree of inflammation and that the expression level of HGF regulates the balance between the HGF/c-Met pathway and the HGF/KAI-1 pathway during both EAE- and CPZ-induced demyelination. Further study will be required to clarify the regulatory mechanism of HGF and its receptor c-Met/KAI-1 in the glial cells and vascular endothelial cells in the CNS during EAE and MS.

## 4. Material and Methods

### 4.1. Animals

Transgenic mice expressing rat HGF in the nervous system (HGF-Tg mice) were backcrossed to C57BL/6J mice, bred, and maintained as previously described [13,17,27]. Age-matched WT C57BL/6J mice served as controls (WT).

### 4.2. Experimental Autoimmune Encephalomyelitis (EAE) Model

EAE induction was performed as previously described [6,20]. In brief, myelin protein peptides including MOG_35–55_ (MEVGWYRSPFSRVVHLYRNGK) were synthesized by Scrum (Tokyo, Japan). Six- to eight-week-old female HGF-Tg+/+, HGF-Tg+/−, and wild-type (WT) mice were immunized s.c. in the flank with an emulsion made of 75 µL of Ag peptide (150 µg of MOG_35–55_) and 75 µL of complete Freund’s adjuvant containing 0.4 mg of heat-inactivated *Mycobacterium tuberculosis* (H37Ra; Difco Laboratories, Franklin Lakes, NJ, USA). Each animal also received 200 ng of pertussis toxin (Sigma Aldrich, St. Louis, MO, USA) through i.p. injection on days 0 and 2 post-immunization. The EAE clinical score was determined and recorded every day as described previously [6,20]: 0, no paralysis; 0.5, stiff tail; 1, limp tail or isolated weakness of gait without limp tail; 2, partial hind limb paralysis; 3, total hind limb or partial hind and front limb paralysis; 4, total hind leg and partial front leg paralysis; and 5, moribund or dead animal [42]. A mean clinical score was assigned to each group using this scale and used for statistical analysis (Mann–Whitney U-test). Histological studies of the lumbar spinal cord were performed to evaluate the EAE-induced pathology.

### 4.3. Cuprizone-Induced Demyelination Model

Cuprizone (CPZ) was purchased from Sigma-Aldrich, and the special chow containing 0.2% CPZ was synthesized (Oriental Yeast Co., Ltd., Chiba, Japan). Female HGF-Tg and WT mice (*n* = 10/treatment) at 8 weeks of age were provided the CPZ chow for 4 weeks [20,43].

### 4.4. Osmium-Maceration SEM Analysis and Immunohistochemistry

In some experiments, scanning electron microscope (SEM) analysis with the osmium-maceration method was applied. We have previously reported that the application of osmium-maceration-based SEM has enabled the detection of early-stage demyelination and more detailed pathological findings related to demyelination and axonal injury in experimental animal models that could not be revealed by conventional transmission electron microscope [6,20,21]. For immunohistochemical study, animals under deep anesthesia (a mixture of medetomidine, midazolam, and butorphanol (0.3 mg/kg, 4.0 mg/kg, and 5.0 mg/kg, respectively), i.p.) were sacrificed and perfused with 0.1 M phosphate buffer (PB, pH 7.4) followed by 4% paraformaldehyde (PFA) in 0.1 M PB [6,20,21]. Spinal cords were removed and immersed in 30% sucrose in 0.1 M PB for 1–2 days. Lumbar spinal cords were then frozen in OCT medium. Frozen 14 µm sections including the lumbar spinal cord were prepared on a cryostat and then stored at −30 °C until use. In brief, the sections were immunostained with anti-Myelin Basic Protein (MBP) antibody (a marker for myelin, 1:1000, clone SMI-94; Biolegend, San Diego, CA, USA [6,21,24]), anti-CC1 antibody (a marker for mature oligodendrocytes, 1:500, Merck Millipore, Darmstadt, Germany [6,21]), anti-GFAP antibody (a marker for astrocytes, 1:1000, Sigma-Aldrich [6,21,24]), anti-Iba 1 antibody (a marker for microglia/macrophage, 1:1000, FUJIFILM WAKO, Osaka, Japan [6,21,24]), anti-F4/80 antibody (a marker for microglia/macrophage, 1:500, BD biosciences, San Jose, CA, USA [6,21,24]), anti-2H3 antibody (a marker for axon, 1:1000, Developmental Studies Hybridoma Bank, Iowa, IA [21,26]), anti-laminin antibody (a marker for endothelial cells, sigma, 1:500 [6,21]), anti-CD31 antibody (a marker for endothelial cells, 1:500, BD biosciences, San Jose, CA [6,21]), anti-c-Met antibody (1:500, Santacruz, Dallas, TX, USA [12,35]), anti-KAI-1 antibody (1:500, Santacruz [12,35]), or anti-excitatory amino acid transporter 2 (EAAT2)/glutamate transporter 1 (GLT-1) antibody (referred to as EAAT2, 1:500, Chemicon/Sigma-Aldrich [12,22,35]). After washing with PBS, the sections were incubated with corresponding secondary antibodies (Alexa Fluor; Molecular Probes/Invitrogen/Thermo Fisher Scientific, Eugen, OR, USA [6,20,21,24]) for 90 min at RT. The sections were further analyzed with a confocal laser microscope (FV-1000D, OLYMPUS, Tokyo, Japan or LSM 780, Carl Zeiss, Oberkochen, Germany) with software (FV10-ASW 3.0, Fluoview, OLYMPUS or Zen, Carl Zeiss) [6,21,24]. The sections after immunostaining were randomly selected, and data from multiple individuals were tabulated and statistically processed. In pathology, cell counting is commonly performed using HE staining [22,23], but a modified similar method by DAPI staining for simple evaluation of inflammatory cells in the CNS, a modified similar method by DAPI staining was adopted in the current study [6,21,24]. It is important to emphasize that although the DAPI staining method does not identify specific cell types, it labels cell nuclei and thus provides a reliable and comprehensive means to conveniently assess whether inflammation is occurring along with the presence of cells. Based on the previous reports, inflammatory foci were characterized by the detection of more than 20 inflammatory cells within the perivascular area surrounding a specified blood vessel [22,23]. ROI measurements were taken on Image J for cell counts and areas of GFAP-positive cells [6,21,24,44]. In some experiments, sections were stained with FluoroMyelin Red (FM; a lipophilic stain for compact myelin, Molecular Probes/Invitrogen/ThermoFisher Scientific) to assess demyelination as described previously [6,20,21,24]. The demyelinated lesion was shown as the FluoroMyelin-negative area in the spinal cord and the corpus callosum. The demyelinated area (% demyelination) in each section was quantified using Image J software (version 1.52-1.54, NIH, Bethesda, MD, USA) [6,20,21,24,44].

### 4.5. Immunoblotting

Animals, deeply anesthetized (a mixture of medetomidine, midazolam, and butorphanol (0.3 mg/kg, 4.0 mg/kg, and 5.0 mg/kg, respectively), i.p.), were sacrificed and perfused with normal saline. Subsequently, only the spinal gray matter was dissected and lysed in cell lysis buffer (cell lytic M lysis buffer; Sigma-Aldrich) [6,21,24]. After determining the protein concentration using the DC protein assay kit (Bio-Rad Laboratories, Hercules, CA, USA), 6 µg of protein extract underwent separation by 15% SDS-PAGE, followed by transfer to a PVDF membrane (Millipore, Darmstadt, Germany). Immunostaining was performed using either anti-synaptophysin antibody (1:1000; abcam, Tokyo, Japan) or anti-GAPDH antibody (1:5000; Sigma-Aldrich). HRP-conjugated secondary antibodies (1:1000, GE Healthcare Systems, Sunnyvale, CA, USA) were applied, and the chemiluminescence assay (ECL) from GE Healthcare Systems was used. Chemiluminescence signals were detected using a Luminoimage Analyzer system (LAS-3000; FUJIFILM, Tokyo, Japan).

### 4.6. Statistical Analysis

Unless otherwise specified, each experimental group consisted of five animals. Histochemical analysis utilized randomly chosen sections from more than three animals per group, analyzed using ImageJ software (version 1.52-1.54, NIH, Bethesda, MD, USA). The results are presented as the mean ± SEM. Unpaired *t*-tests were employed for pairwise comparisons, and one-way ANOVA followed by Bonferroni tests was used for multiple comparisons. Statistical significance was set at *p* < 0.05 [6,21,24].

## 5. Conclusions

Our results suggest that HGF exerts a pleiotropic protective effect on pathways involved in reducing inflammatory demyelination and axonal injury while promoting neuroprotective mechanisms. Although there are various therapies aimed at reducing inflammation, few current drugs are disease-modifying, particularly for progressive MS. The studies presented here demonstrate a potential novel therapy targeting HGF, which offers significant neuroprotective effects. Furthermore, in two distinct models of MS evaluated for the efficacy of HGF treatment, HGF not only attenuated demyelination and protected axons but also improved behavioral outcomes in paralysis. Thus, HGF emerges as a promising therapeutic candidate for MS.

## Figures and Tables

**Table 1:** The effect of HGF on the progression of EAE.

	Incidence	Mortality	Day of Onset	Mean Max Grade	Grade at the Peak	
					Max	Min
Wild type	23/23	-	12.3 ± 0.3	2.6 ± 0.56	3.5	2.0
HGF-Tg +/−	23/23	-	12.8 ± 0.8	2.3 ± 0.40	3.0	1.0
HGF-Tg +/+	22/24	-	15.5 ± 1.8 **	1.0 ± 0.8 **	2.5	0.0

** *p* < 0.01 vs. WT mice.

## Data Availability

All data produced for this manuscript are available from the lead contact (Y.B.) upon reasonable request.

## Abbreviations

BBB: blood–brain barrier; CNS, central nervous system; DAPI, 4′,6-diamidino-2-phenylindole; EAE, experimental autoimmune encephalomyelitis; GFAP, glial fibrillary acid protein; Iba-1, ionized calcium-binding adapter molecule 1; MBP, myelin basic protein; MOG, myelin oligodendrocyte glycoprotein; MS, multiple sclerosis; OLs, oligodendrocytes; OPC, oligodendrocyte precursor cell; PB, phosphate buffer; PFA, paraformaldehyde.
